# Hearing Loss and Cognitive Decline: Causal Evidence

**DOI:** 10.1093/geroni/igaa057.2924

**Published:** 2020-12-16

**Authors:** Justin Golub, Rahul Sharma, Alex Chern, Brady Rippon, Adam Ciarlegio, Nicole Schupf, Adam Brickman, José Luchsinger

**Affiliations:** 1 NewYork-Presbyterian Hospital/Columbia University Irving Medical Center, New York City, New York, United States; 2 Columbia University Irving Medical Center, New York, New York, United States; 3 Columbia University, New York, New York, United States; 4 George Washington University, Washington, District of Columbia, United States; 5 Columbia University Medical Center, New York, New York, United States

## Abstract

Age-related hearing loss (HL) has recently been associated with cognitive decline, dementia, and changes in brain structure. HL has near universal prevalence in later life (involving 80% of those over 80 years old) and is rarely treated (under 20% in the same age group). Dementia is also common in later life, carrying staggering societal implications, including a worldwide cost of over $605 billion/year. Research establishing an association between HL and cognition has included cross-sectional and prospective studies. New data show a link not only in the earliest stages of HL but also in hearing ranges still considered normal. The current evidence for an independent association between HL and impaired cognition, including a possible causal connection, will be reviewed. Plausible mechanistic pathways will be discussed with an emphasis on brain imaging biomarkers of dementia. Finally, future avenues for research and policy change will be proposed.

